# *Reevesia* in a Warmer World: Mapping the Habitat Suitability of Its Two Representative Species (*R. pubescens* and *R. thyrsoidea*) in China

**DOI:** 10.1002/ece3.72934

**Published:** 2026-01-12

**Authors:** Xuanqi Liu, Xia Meng, Minqiao Li, Zeyu Qin, Chen Li, Huasheng Huang

**Affiliations:** ^1^ School of Geography and Planning Sun Yat‐sen University Guangzhou China; ^2^ Carbon‐Water Observation and Research Station in Karst Regions of Northern Guangdong, School of Geography and Planning Sun Yat‐sen University Guangzhou China

**Keywords:** biodiversity, biological conservation, climate change, Malvaceae, MaxEnt, *Reevesia*, species distribution models

## Abstract

Climate change is altering the spatial distribution of species' suitable habitats. Congeneric species may exhibit divergent responses to climate change due to differences in their niches. A clear understanding of these differences is essential for the development of effective and targeted biodiversity conservation strategies. Here, we use the optimized maximum entropy model (MaxEnt) to simulate the current distributions of two congeneric species, *Reevesia pubescens* and *R. thyrsoidea*, and project their future distributions under different climate scenarios across China. Despite their close taxonomic relationship, the two species show distinct climatic preferences and distributional patterns. *R. pubescens* primarily occupies montane regions in southwestern China. Its distribution is most influenced by precipitation and temperature during the driest quarter, as well as precipitation seasonality. In contrast, *R. thyrsoidea* prefers lowland coastal areas in southeastern China. Its distribution responds more strongly to temperature stability and wet‐season precipitation. Meanwhile, for both species, climatic factors, rather than terrain, are the dominant forces shaping their current distributions. Under future climate scenarios, both species are projected to expand their suitable habitats, although with different magnitudes and directions. *R. pubescens* shows moderate northward expansion under the low‐emission scenario. However, under the high‐emission scenario, this expansion is constrained, likely due to thermal limitations. *R. thyrsoidea* demonstrates robust expansion under both scenarios. Spatial overlap between the two species is expected to increase, especially by 2081–2100, suggesting potential areas for joint conservation. Our results highlight the importance of species‐specific ecological strategies and underscore the role of climate adaptation in shaping future distributions, and provide valuable insights for conservation planning under climate change.

## Graphical abstract

We mapped the habitat suitability of *Reevesia pubescens* and *R. thyrsoidea* in China. The two species show distinct climatic preferences and ecological zones. Climatic factors, rather than terrain, mainly shape their distributions.

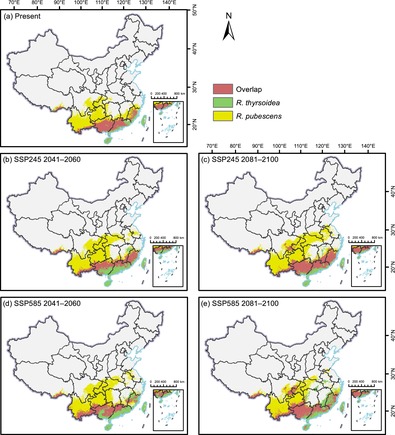

## 1 Introduction

Climate change has become one of the most pressing environmental challenges of the 21st century. Global surface temperatures have already increased by approximately 1.2°C above pre‐industrial levels (Intergovernmental Panel on Climate Change (IPCC) 2021). This warming trend is expected to intensify, leading to more frequent and severe extreme weather events, rising sea levels, and disruptions to both ecological systems and human societies (Bellard et al. 2012; Osman et al. 2021). Species are particularly sensitive to climate shifts, and many have already exhibited noticeable changes in their geographic ranges in response to these environmental stresses (Wiens and Zelinka 2024). Some are migrating toward higher altitudes or latitudes in search of more favorable conditions, whereas others are suffering from habitat loss or even local extinction due to reduced climate suitability (Wang et al. 2024). A comprehensive understanding of species' potential geographic distributions under different climate change scenarios is essential. It provides a critical foundation for effective biodiversity conservation, ecosystem management, and long‐term ecological planning.

*Reevesia* is a genus in the family Malvaceae, comprising 14 species (Plants of the World Online [POWO], https://powo.science.kew.org/). It was previously classified in Sterculiaceae (Geng et al. 2024). The genus displays a classic East Asia‐Central America disjunct distribution. All species, with the exception of *R. clarkii* (native to Central America), are found in Southeast Asia, and China serves as a major center of diversity (POWO, https://powo.science.kew.org/). Several *Reevesia* species are valued for their ecological and economic contributions. They are cultivated as ornamental plants due to their large corymbose inflorescences, fragrant white flowers, and esthetically pleasing foliage structure (iPlant database, http://www.iplant.cn). The wood is dense and straight‐grained, which makes it suitable for furniture and plywood production. The bark contains strong fibers used for rope‐making, sack weaving, and papermaking. Moreover, the seeds of some species are used for essential oil extraction (Bayer and Kubitzki 2003; Chang et al. 2013; Quan et al. 2019). Recent phytochemical studies have identified bioactive compounds in *R. formosana* (a synonym of *R. thyrsoidea*). These compounds show promising cytotoxic activity, suggesting potential applications in anticancer drug development (Chang et al. 2013, 2017; Hsiao et al. 2016).

Beyond these economic and pharmacological values, chloroplast genomic evidence reveals its phylogenetic distinctiveness and provides key molecular resources for species delimitation and evolutionary studies (Geng et al. 2024; Quan et al. 2019; Zhang et al. 2022). These findings highlight its evolutionary significance and conservation urgency. Many *Reevesia* species are regionally endemic, with narrow distributions and habitat specialization that make them vulnerable to habitat loss and climate change. For example, *R. pycnantha* is restricted to subtropical hillsides and forest edges in southeastern China, where habitat degradation and environmental change could lead to rapid population decline (Zhang et al. 2022). As global warming accelerates, these species may face intensified thermal stress, range fragmentation, and ecological mismatch with their current habitats, threatening their long‐term survival. Despite its ecological and economic significance, the current and future distributions of *Reevesia* species under climate change remain poorly understood. This is particularly concerning given the mounting evidence that climate change is altering species biogeographic ranges and reshaping ecological interactions.

Species distribution models (SDMs) simulate the relationship between species occurrences and environmental variables to estimate the potential geographic distribution of species (Elith and Leathwick 2009). These models have become essential tools for predicting species suitable habitats under current and future climatic conditions (e.g., Liu et al. 2025). For instance, the maximum entropy model (MaxEnt) (Phillips et al. 2006), random forest (RF) (Breiman 2001), generalized linear models (GLM) (Nelder and Wedderburn 1972), and generalized additive models (GAM) (Hastie and Tibshirani 1987) have been widely used in biogeographic studies. Among these, MaxEnt is particularly popular due to its excellent predictive performance when only presence data are available (Xiao et al. 2024; Zhao et al. 2024). The MaxEnt algorithm estimates the probability distribution of maximum entropy; that is, it identifies the most uniform distribution permissible under the constraint that the expected value of each environmental variable matches its empirical mean across known occurrence locations (Phillips et al. 2006). This approach enables robust predictions even with limited or biased data (Phillips and Dudík 2008). As a result, MaxEnt has been widely applied in species conservation, biological invasion monitoring, and climate change impact assessments (Xiao et al. 2024; Zhao et al. 2024).

**FIGURE 1:**
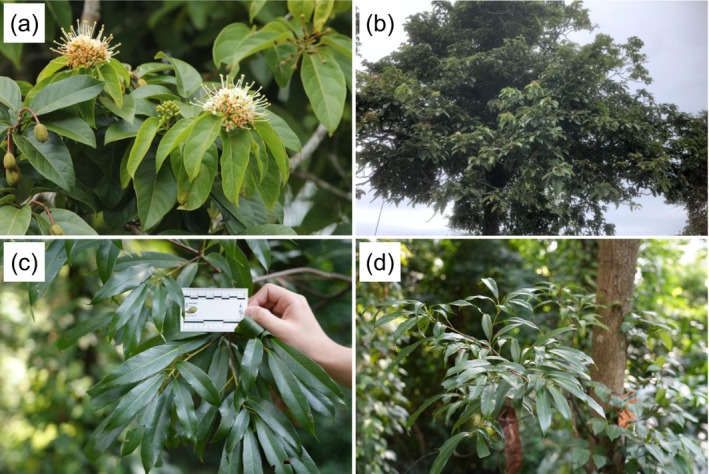
(a, b) *Reevesia pubescens* (observed in Thailand by Woraphot Bunkhwamdi; licensed under http://creativecommons.org/licenses/by‐nc/4.0/); (c, d) *R. thyrsoidea* (photographed by Xuanqi Liu at South China National Botanical Garden, Guangzhou, China).

Here, we focus on two representative species of *Reevesia*: *R. pubescens* Mast. and *R. thyrsoidea* Lindl. (Figure 1), which together account for over 80% of known occurrence records in the genus. They occupy distinct biogeographic and climatic zones: *R. pubescens* is mainly distributed in southwestern (SW) China, whereas *R. thyrsoidea* occurs predominantly in Guangdong and Guangxi provinces of South China. Such congeneric divergence is common in plants and may result in contrasting responses to environmental change (Anacker and Strauss 2014; Hosseini et al. 2024). However, few have explicitly examined how such divergence affects the responses of species to future climate change. Understanding these differential responses is essential for predicting shifts in biodiversity and ecosystem stability under global warming (Wang et al. 2024).

In this study, we use an optimized MaxEnt model to simulate the potential distributions of the two species under present and future climate conditions. Our aim is to provide a comparative perspective on species‐level responses within *Reevesia*, thereby offering insights into adaptive conservation strategies. Specifically, we address the following key questions: (1) What are the current distribution patterns of *R. pubescens* and *R. thyrsoidea*, and which environmental factors most strongly determine their habitat suitability? (2) How are their suitable habitats projected to shift geographically under low‐ and high‐emission climate scenarios? (3) To what extent will their future distributions overlap, and what implications does this have for conservation planning?

## 2 Materials and Methods

### 2.1 Species Occurrence Data

**FIGURE 2:**
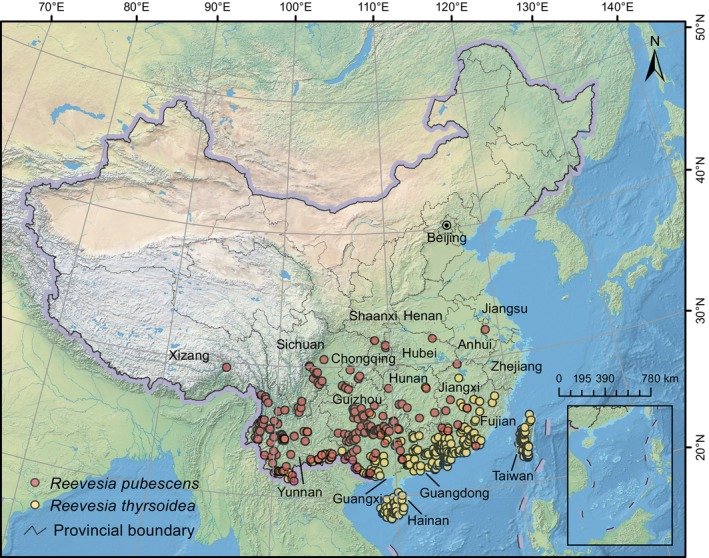
Current distribution with cleaned occurrence data of *Reevesia pubescens* and *R. thyrsoidea* in China. The base map was produced in accordance with the standard map issued by the Ministry of Natural Resources of China (Map Review Number: GS(2019)1822), and the boundaries have not been modified.

To obtain comprehensive and reliable occurrence data for SDMs, we compiled all available records within China from the Global Biodiversity Information Facility (GBIF, http://www.gbif.org; GBIF.org 2025) and the National Plant Specimen Resource Center (NPSRC, http://www.cvh.ac.cn/). Erroneous and imprecise records were removed using the R CoordinateCleaner package (Zizka et al. 2019). Additionally, the reliability of each occurrence point was further validated based on taxonomic and geographic information from the Flora Reipublicae Popularis Sinicae (FRPS, https://www.iplant.cn/) and POWO (https://powo.science.kew.org/). To reduce spatial autocorrelation, we applied spatial filtering using ENMTools (Warren et al. 2021), with only one occurrence per 30 arc‐seconds (~1 km) grid cell. After all filtering steps, a total of 233 occurrences for *R. pubescens* (Supporting Information, Table S1) and 415 for *R. thyrsoidea* (Supporting Information, Table S2) were retained for modeling (Figure 2). This study restricts species distribution modeling to China, as the vast majority of verified occurrence records are concentrated there. Although both species have been reported from neighboring regions (e.g., Vietnam, Thailand, and Laos), these records are few and spatially sparse. Incorporating such limited data into model calibration could increase sampling bias and reduce prediction robustness, particularly at regional scales (Baker et al. 2022). Consequently, constraining the modeling extent to China provides a more robust and ecologically meaningful characterization of species–environment relationships.

### 2.2 Environmental Variables

We initially selected 22 environmental variables that could potentially influence the distribution of *R. pubescens* and *R. thyrsoidea*. These include 19 bioclimatic variables obtained from the WorldClim database version 2.1 (http://www.worldclim.org), as well as elevation data. In addition, slope and aspect were derived using the 3D Analyst toolbox in ArcGIS 10.8.1. All data had a spatial resolution of 30 arc‐seconds. To minimize multicollinearity among predictors, we calculated Pearson's correlation coefficients for the 19 bioclimatic variables (Figure S1). Variables with pairwise correlation coefficients |*r*| ≥ 0.8 were considered highly collinear. From each correlated pair, only one variable was retained. The selection was based on ecological relevance and preliminary model performance. Topographic variables (elevation and slope) were retained a priori and were not excluded based on their correlations with climatic variables. This is because topography contributes independent ecological information, particularly by capturing terrain‐related influences on local climate, hydrological, and microenvironmental conditions that are not fully represented by macroclimatic variables (Badgley et al. 2017; Dobrowski 2011; Guisan and Zimmermann 2000). During preliminary model testing, aspect was included as a predictor, and its contribution was evaluated using multiple diagnostic approaches within the MaxEnt framework. The results showed that aspect contributes negligibly to model performance (Supporting Information, Figures S2–S6). Therefore, aspect was excluded from further analysis. This resulted in a total of 11 environmental variables for species distribution modeling, including elevation, slope, and nine bioclimatic variables (Table 1).

**TABLE 1:** The 11 Environmental factors for MaxEnt modeling.

Variables	Description	Units
Bio1	Annual mean temperature	°C
Bio2	Mean diurnal range (mean of monthly (max temp−min temp))	°C
Bio3	Isothermality (bio2/bio7) (×100)	/
Bio7	Temperature annual range (bio5 − bio6)	°C
Bio9	Mean temperature of driest quarter	°C
Bio10	Mean temperature of warmest quarter	°C
Bio13	Precipitation of wettest month	mm
Bio15	Precipitation seasonality (coefficient of variation)	/
Bio17	Precipitation of driest quarter	mm
Elev	Elevation, height relative to datum	m
Slope	Angle of inclination of the slope	°

For future climate projections, we selected the BCC‐CSM2‐MR global climate model from the Coupled Model Intercomparison Project Phase 6 (CMIP6), available via WorldClim v2.1. This model has demonstrated robust performance in simulating climatic conditions in China (Wu et al. 2019). Future climate data were obtained for two time periods (2041–2060 and 2081–2100) under two Shared Socioeconomic Pathways (SSPs): SSP245 and SSP585. SSP245 represents a stabilization pathway with moderate greenhouse gas emissions and a sustainability‐oriented trajectory. In contrast, SSP585 depicts a high‐emission scenario characterized by fossil‐fuel‐driven development and more severe projected climate changes.

### 2.3 MaxEnt Model

We used the MaxEnt model to simulate the potential distribution of the two *Reevesia* species based on presence‐only occurrence data. MaxEnt estimates the probability distribution of suitable habitats by maximizing entropy, constrained by environmental conditions at known occurrence locations (Phillips et al. 2006). To optimize model complexity and predictive performance, we used the R ‘ENMeval’ package to fine‐tune two key parameters: the regularization multiplier (RM) and feature combinations (FC) (Kass et al. 2021). Specifically, we adjusted the Regularization RM and FC to balance model complexity and predictive accuracy. We tested eight RM values (0.5–4.0, at 0.5 intervals) and five FC settings (L, LQ, LQH, LQHP, and LQHPT). L, Q, H, P, and T are linear, quadratic, hinge, product, and threshold features, respectively. We evaluated model performance using the corrected Akaike information criterion (AICc) and selected the optimal parameter set based on the minimum delta.AICc (delta.AICc = 0). delta.AICc represents the difference in AICc between each candidate model and the model with the lowest AICc; lower values indicate better model parsimony. The best‐performing combination was RM = 1.5 and FC = LQHPT.

We applied 10‐fold cross‐validation to assess the robustness of each model. For each replicate, 10,000 background points were randomly drawn from the study area. Final predictions were averaged across the 10 model runs to reduce random variance. We used the receiver operating characteristic (ROC) curve to evaluate model accuracy. The ROC curve plots the true positive rate against the false‐positive rate across threshold values. The area under the curve (AUC) reflects overall model performance; AUC values range from 0 to 1, with values closer to 1 indicating higher discriminatory power (Shabani et al. 2018).

Habitat suitability values predicted by MaxEnt were categorized into four levels based on the probability of occurrence (*p*): unsuitable (*p* < 0.2), low suitable (0.2 ≤ *p* < 0.4), moderately suitable (0.4 ≤ *p* < 0.6), and extremely suitable (*p* ≥ 0.6) (Wang et al. 2024). For binary classification and overlap analysis, a conservative threshold of *p* = 0.2 was used to define suitable versus unsuitable areas. We then mapped and quantified the extent of each suitability class and used the Mean Center tool in ArcGIS 10.8.1 to calculate spatial shifts in habitat centroids under future climate scenarios. This approach enabled us to visualize and interpret potential range dynamics and spatial overlap between the two species.

## 3 Results

### 3.1 Current Distribution

The optimized MaxEnt models for both species exhibited excellent predictive performance, as indicated by high AUC values of 0.952 for *R. pubescens* and 0.968 for *R. thyrsoidea*. These values suggest a strong ability to distinguish between suitable and unsuitable habitats for each species under current climatic conditions. These results were achieved following systematic parameter optimization and represent a notable improvement over models calibrated with default settings.

**FIGURE 3:**
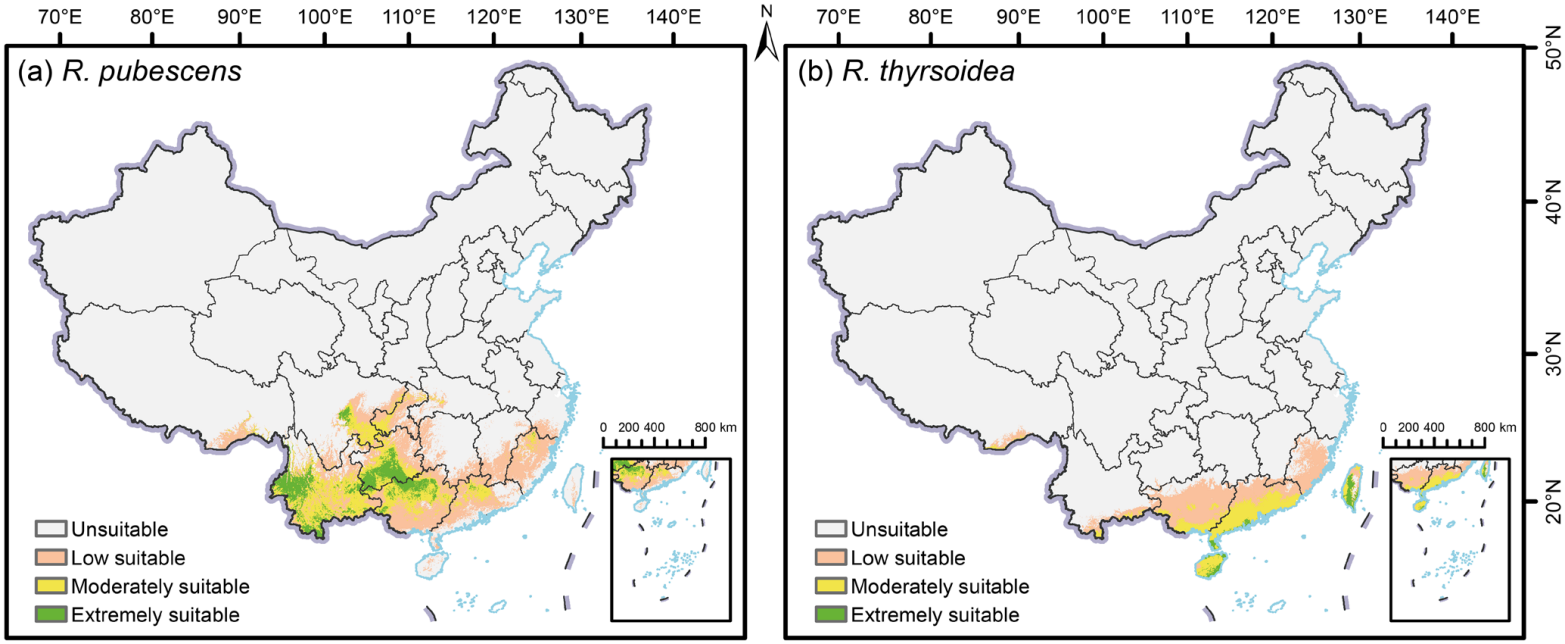
Current projected distributions of *Reevesia pubescens* (a) and *R. thyrsoidea* (b) using MaxEnt model.

The predicted current distributions reveal clear spatial differentiation between the two species, reflecting distinct ecological preferences and habitat requirements (Figure 3). For *R. pubescens* , extremely suitable habitats are concentrated in SW China, particularly in western Yunnan, southern Guizhou, and northwestern Guangxi, with smaller patches observed in central Sichuan. These extremely suitable areas cover 14.92 × 10^4^ km^2^. Moderately suitable (33.30 × 10^4^ km^2^) and low suitable (63.19 × 10^4^ km^2^) areas are more extensively distributed, spanning eastern Yunnan, northern Guizhou, large parts of Guangxi, western Guangdong, and Fujian. Additional fragmented patches occur in southeastern Sichuan, Chongqing, Hunan, Jiangxi, and southern Tibet. In contrast, *R. thyrsoidea* displays a distinct preference for the southeastern coastal regions of China. Extremely suitable areas (3.35 × 10^4^ km^2^) are primarily located along the southern coast of Guangxi and southwestern Guangdong, as well as the eastern and southern coasts of Hainan and central Taiwan. Moderately suitable (15.77 × 10^4^ km^2^) and low suitable (30.47 × 10^4^ km^2^) areas extend broadly across Guangdong, Guangxi, Fujian, Hainan, and Taiwan. Small patches of low suitable habitat are also found in southern Tibet and southern Yunnan. These contrasting distribution patterns reflect the divergent climatic preferences of the two species: *R. pubescens* is associated with montane, subtropical inland environments, whereas *R. thyrsoidea* is more strongly adapted to warm, humid coastal, and insular habitats.

### 3.2 Variable Importance and Response Curves

We evaluated the relative influence of environmental variables for *R. pubescens* and *R. thyrsoidea* using both permutation importance and response curves. Permutation importance was quantified as the reduction in model performance when the values of a given variable were randomly permuted while all other variables were held constant; larger performance declines indicate a greater contribution of that variable to the model. Additionally, jackknife tests of variable importance for both species distribution models are provided in Supporting Information (Figures S8–S13), providing complementary information on variable independence, model gain, and contribution.

**FIGURE 4:**
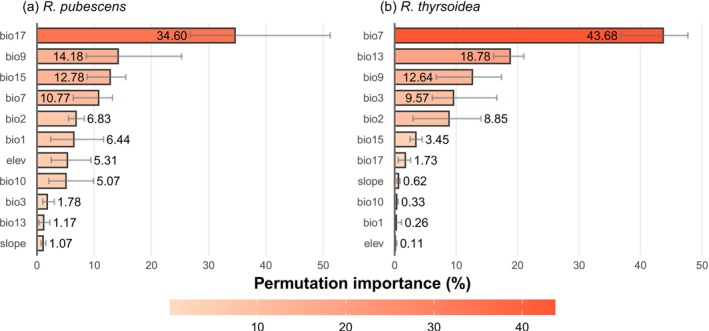
The importance of different environmental variables for *Reevesia pubescens* (a) and *R. thyrsoidea* (b). The full name and unit of each variable are provided in Table 1. The gray short lines show the fluctuation range from multiple random simulations.

The key predictors varied notably between the two species, suggesting distinct ecological requirements (Figure 4). For *R. pubescens* , the most influential variable was precipitation of driest quarter (bio17), underscoring the importance of water availability during dry seasonal periods. In contrast, *R. thyrsoidea* was most strongly influenced by temperature annual range (bio7), indicating a greater sensitivity to thermal variability throughout the year. Notably, mean temperature of driest quarter (bio9) ranked highly in importance for both species, suggesting that thermal conditions during the driest months are broadly relevant to their distributions. Additional important variables for *R. pubescens* included precipitation seasonality (bio15) and temperature annual range (bio7), highlighting the role of climatic variability. For *R. thyrsoidea*, precipitation of wettest month (bio13) and isothermality (bio3) also made significant contributions. In contrast, topographic variables such as elevation and slope exhibited low permutation importance in both models, suggesting a subordinate role relative to bioclimatic factors.

**FIGURE 5:**
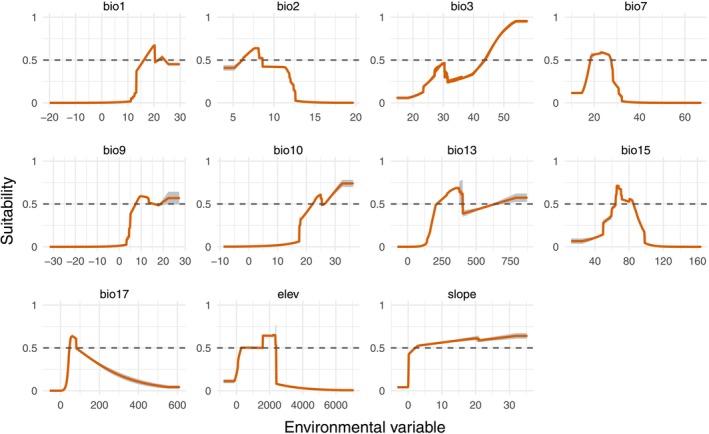
Environmental variable response curve for *Reevesia pubescens*. The full name and unit of each variable are provided in Table 1. The light gray background indicates the fluctuation range of multiple random simulations.

The response curves provided further insight into each species' environmental preferences. For *R. pubescens* , the suitable annual mean temperature (bio1) is around 20°C, with a preferred mean diurnal range (bio2) of approximately 7.5°C (Figure 5). The species favors isothermality (bio3) values above 55, with a smaller peak around 28. It is associated with a temperature annual range (bio7) near 25°C, and mean temperatures of the driest (bio9) and warmest (bio10) quarters exceeding 10°C and 30°C, respectively. Optimal precipitation values include around 350 mm during the wettest month (bio13), a precipitation seasonality (bio15) value near 70, and approximately 50 mm during the driest quarter (bio17). Suitable habitats are typically found at elevations (elev) around 2000 m, while slope does not appear to have a clear influence.

**FIGURE 6:**
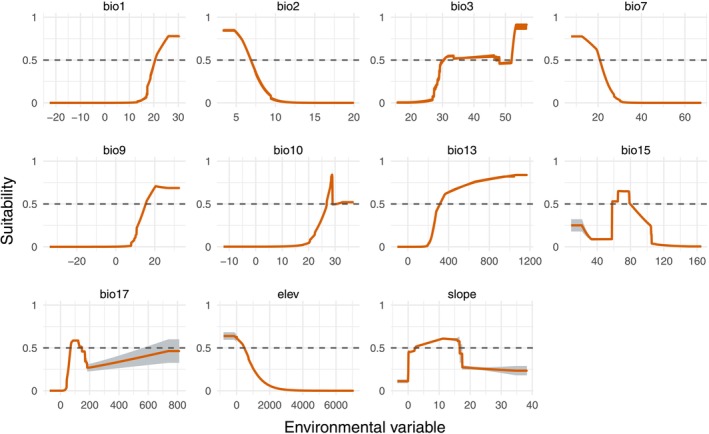
Environmental variable response curve for *Reevesia thyrsoidea*. The full name and unit of each variable are provided in Table 1. The light gray background indicates the fluctuation range of multiple random simulations.

In comparison, *R. thyrsoidea* prefers higher annual mean temperatures (bio1), generally above 26°C, and a mean diurnal range (bio2) below 5°C (Figure 6). It also favors isothermality (bio3) values above 55 and a temperature annual range (bio7) below 15°C. Suitable conditions include a mean temperature of the driest quarter (bio9) above 20°C and of the warmest quarter (bio10) near 30°C. The species is associated with precipitation over 400 mm in the wettest month (bio13), a seasonality index (bio15) around 70, and about 100 mm in the driest quarter (bio17). Preferred habitats occur at elevations (elev) near 500 m and on gentle slopes between 0° and 15°.

### 3.3 Future Distribution and Centroid Shift

**FIGURE 7:**
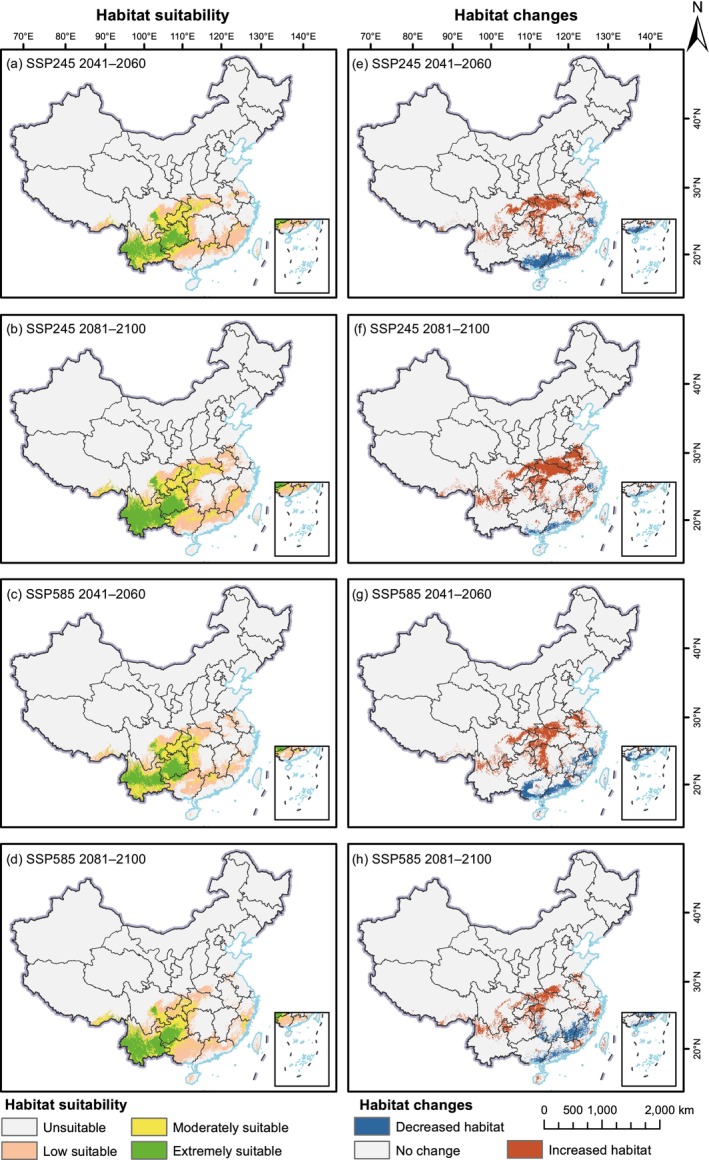
Distribution and changes of future suitable areas compared to its current distribution for *Reevesia pubescens* under different scenarios. (a–d) show future suitable areas, and (e–h) illustrate future habitat suitability changes relative to the current distribution.

**FIGURE 8:**
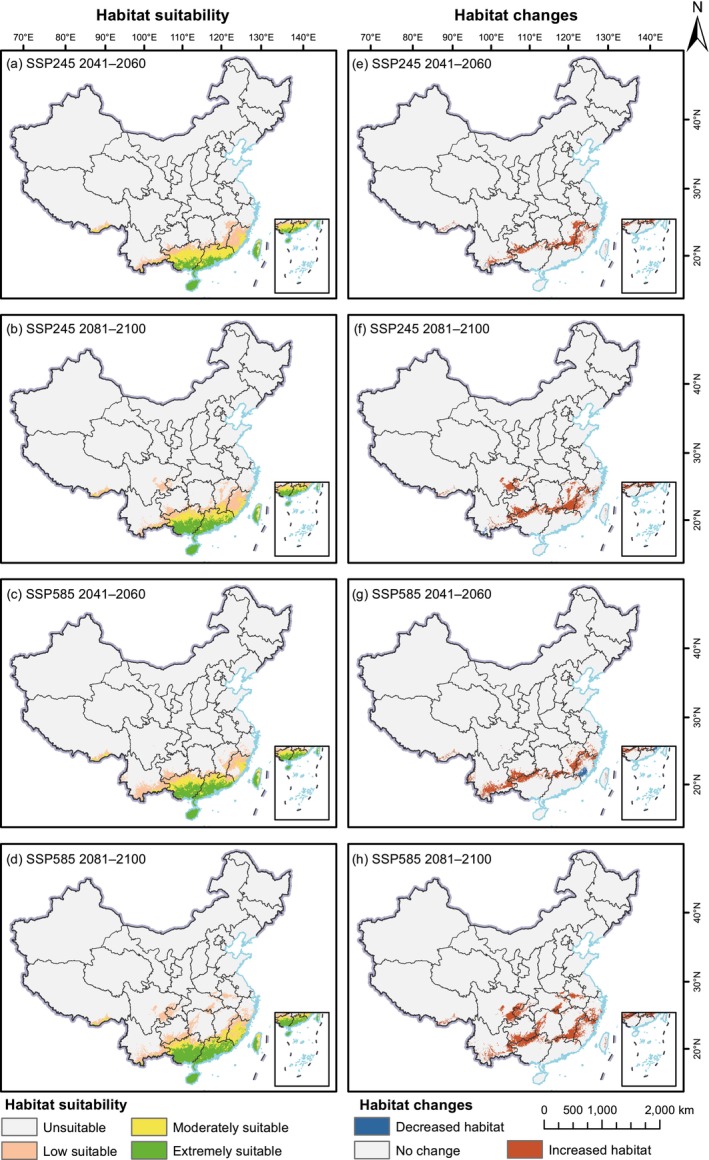
Distribution and changes of future suitable areas compared to its current distribution for *Reevesia thyrsoidea* under different scenarios. (a–d) show future suitable areas, and (e–h) illustrate future habitat suitability changes relative to the current distribution.

Using the MaxEnt model, we projected the future potential distributions of *R. pubescens* and *R. thyrsoidea* under different climate scenarios (in 2041–2060 and 2081–2100 under SSP245 and SSP585) (Figures 7 and 8). The area of each suitable zone and the percentage of its change are shown in Table 2. Overall, highly suitable habitats for *R. pubescens* are expected to remain concentrated in Yunnan and Guizhou. Moderately and lowly suitable areas are projected to expand more broadly across western Sichuan, Chongqing, Hubei, Guangxi, Guangdong, and Fujian. Compared to the current total suitable area of 111.42 × 10^4^ km^2^, the overall habitat suitable area for *R. pubescens* is projected to increase under all future climate scenarios. Under the SSP245 scenario, the total suitable area increases to 133.66 × 10^4^ km^2^ during 2041–2060 (19.96%) and further expands to 156.46 × 10^4^ km^2^ by 2081–2100 (40.42%). Under the high‐emission SSP585 scenario, the total suitable area reaches 132.21 × 10^4^ km^2^ (18.66%) in 2041–2060 and 123.56 × 10^4^ km^2^ (10.89%) in 2081–2100. Among all suitability classes, the extremely suitable area exhibits the most pronounced expansion. This area increases from 14.92 × 10^4^ km^2^ at present to 29.65 × 10^4^ km^2^ under SSP245 by 2041–2060, representing a growth of 98.72%, and further expands to 39.97 × 10^4^ km^2^ (167.88%) by 2081–2100. A similar trend is observed under SSP585, with extremely suitable areas reaching 30.06 × 10^4^ km^2^ (101.44%) and 29.77 × 10^4^ km^2^ (99.48%) during the two future periods, respectively. The moderately suitable area also shows considerable increases, with growth rates of 28.46% and 41.94% under SSP245, and 31.55% and 4.02% under SSP585 for the two future periods, respectively. In contrast, the low suitable area shows a clear increase only under SSP245 in 2081–2100 (9.53%), while it declines under all other scenarios.

**TABLE 2:** The different‐level suitable area for the present and future (10^4^ km^2^), and the areal differences that were calculated compared with the present suitable area (%).

Species	Period	Extremely suitable		Moderate suitable		Low suitable		Total	
		Area	Change	Area	Change	Area	Change	Area	Change
*R. pubescens*	Modern	14.92	/	33.30	/	63.19	/	111.42	/
	SSP245 2041–2060	29.65	98.72	42.78	28.46	61.23	−3.11	133.66	19.96
	SSP245 2081–2100	39.97	167.88	47.27	41.94	69.21	9.53	156.46	40.42
	SSP585 2041–2060	30.06	101.44	43.81	31.55	58.34	−7.69	132.21	18.66
	SSP585 2081–2100	29.77	99.48	34.64	4.02	59.15	−6.40	123.56	10.89
*R. thyrsoidea*	Modern	3.35	/	15.77	/	30.47	/	49.59	/
	SSP245 2041–2060	16.58	394.51	23.69	50.20	24.57	−19.37	64.84	30.74
	SSP245 2081–2100	23.59	603.63	19.32	22.50	28.22	−7.37	71.14	43.44
	SSP585 2041–2060	23.11	589.21	18.40	16.66	27.57	−9.52	69.08	39.29
	SSP585 2081–2100	29.53	780.56	19.55	23.92	34.68	13.84	83.76	68.88

Compared with the current distribution, the future suitable range of *R. pubescens* is projected to exhibit a clear trend of northward expansion and southward contraction (Figure 7). Under the SSP245 scenario, the expansion of suitable habitats is mainly concentrated in northern and western Hubei, southern Henan, and central to northern Anhui. Under the SSP585 scenario, the newly gained suitable areas are primarily located in northern and western Hubei as well as western Hunan. Overall, the northward expansion of suitable habitats is more pronounced under the SSP245 scenario. In contrast, habitat loss is primarily concentrated in the southern coastal regions of Guangxi and Guangdong. Under the SSP585 scenario, this southward contraction becomes even more pronounced, with substantial reductions also projected in southern Hunan, southern Jiangxi, and parts of Fujian. In general, the SSP245 scenario appears more favorable for the future survival of *R. pubescens* than SSP585, especially in terms of total suitable area and the expansion of extremely suitable area.

Overall, in future climate scenarios, extremely suitable areas for *R. thyrsoidea* are projected to remain concentrated in southern Guangdong and Guangxi, as well as Hainan and western Taiwan (Figure 8). Moderately and lowly suitable areas are mainly distributed across northern Guangdong and Guangxi, Fujian, and southeastern Yunnan.

Relative to its current total suitable area of 49.59 × 10^4^ km^2^, *R. thyrsoidea* shows consistent expansion in suitable habitat under all projected climate conditions. Under SSP245, the total suitable area reaches 64.84 × 10^4^ km^2^ in 2041–2060 (30.74%) and expands further to 71.14 × 10^4^ km^2^ in 2081–2100 (43.44%). Under the SSP585 scenario, even larger gains are predicted, with areas increasing to 69.08 × 10^4^ km^2^ (39.29%) and 83.76 × 10^4^ km^2^ (68.88%) across the same time periods. The most remarkable growth occurs within the extremely suitable category. From a current extent of only 3.35 × 10^4^ km^2^, this area expands to 16.58 × 10^4^ km^2^ (394.51%) under SSP245 in 2041–2060 and reaches 23.59 × 10^4^ km^2^ (603.63%) in 2081–2100. Under SSP585, the increases are even more pronounced, reaching 23.11 × 10^4^ km^2^ (589.21%) in 2041–2060 and 29.53 × 10^4^ km^2^ (780.56%) in 2081–2100. Moderately suitable habitats also increase substantially, with growth rates of 50.20% and 22.50% under SSP245, and 16.66% and 23.92% under SSP585. In contrast, low suitable areas show less consistent trends—most scenarios suggest a slight decline, except for SSP585 in 2081–2100, which projects a 13.84% increase.

Unlike *R. pubescens* , the future distribution of *R. thyrsoidea* shows a strong tendency toward northward expansion, with very limited range contraction. New areas of suitability are expected to emerge in northern Guangdong and Guangxi, southeastern Yunnan, eastern Jiangxi, and Fujian. In 2081–2100, under both SSP245 and SSP585, the species' potential range is projected to expand into southeastern Sichuan and parts of southwestern Chongqing. Notably, the most extensive northward expansion occurs under SSP585 in the late 21st century. Only minor habitat loss is observed, confined to a small area in southern Fujian during 2041–2060 under SSP585. Overall, the future outlook for *R. thyrsoidea* is favorable, especially under the high‐emission SSP585 scenario, which promotes more considerable expansion in both the extent and quality of suitable habitats.

Systematic comparisons between future time periods (2041–2060 vs. 2081–2100) and emission scenarios (SSP245 vs. SSP585) reveal distinct long‐term distributional trends for both species. For *R. pubescens* , the expansion of suitable habitats is more pronounced in the mid‐21st century, particularly under SSP245, but slows or partially reverses by the late 21st century under SSP585, indicating emerging constraints under extreme warming conditions. In contrast, *R. thyrsoidea* exhibits a consistent and progressively intensified expansion from the mid to late 21st century under both scenarios, with the largest gains occurring under SSP585. These patterns highlight the contrasting temporal trajectories and differential sensitivities to emission scenarios between the two species.

**FIGURE 9:**
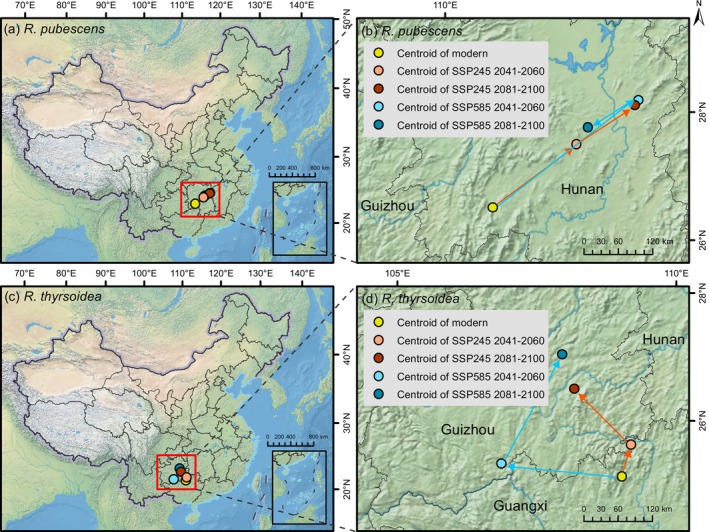
Spatial distribution and projected shifts of suitable habitat centroids for *Reevesia pubescens* (a, b) and *R. thyrsoidea* (c, d) under different climate change scenarios. (b) and (d) provide enlarged views of the centroid movement paths. Red lines indicate centroid shifts under SSP245, while blue lines represent those under SSP585.

We merged the extremely suitable, moderately suitable, and low suitable areas to calculate the centroids of suitable habitats for *R. pubescens* and *R. thyrsoidea* under various future climate scenarios (Figure 9, Table 3). Analysis of centroids dynamics reveals that the suitable habitat centroids of *R. pubescens* consistently remain within Hunan Province. Under SSP245, the centroid gradually shifts northeastward, with further movement in the same direction by 2081–2100. In contrast, under SSP585, the centroid initially shifts northeastward during 2041–2060, but then reverses direction and shifts southwestward by the end of the century. For *R. thyrsoidea*, the current centroid is located across Guangxi and Guizhou. Under both climate scenarios, the suitable habitat centroids exhibit a general northward shift, moving from Guangxi to Guizhou. Under SSP245, the shift first occurs toward the northeast, followed by a northwestward movement. Conversely, under SSP585, the centroid initially shifts northwest and then turns northeast in the later period.

**TABLE 3:** Latitude and longitude of habitat suitability centroids under different climate change scenarios, along with the distance shifted compared to the previous time period.

Period	*R. pubescens*		*R. thyrsoidea*	
	Coordinates	Distance (km)	Coordinates	Distance (km)
Modern	110.67° E, 26.74° N	/	108.90° E, 25.17° N	/
SSP245 2041–2060	112.23° E, 27.65° N	184.00	109.08° E, 25.66° N	57.97
SSP245 2081–2100	113.33° E, 28.18° N	122.76	108.12° E, 26.57° N	139.32
SSP585 2041–2060	113.40° E, 28.25° N	317.33	106.81° E, 25.42° N	212.37
SSP585 2081–2100	112.46° E, 27.89° N	101.10	107.92° E, 27.12° N	218.74

### 3.4 Overlapping Distribution Areas

**FIGURE 10:**
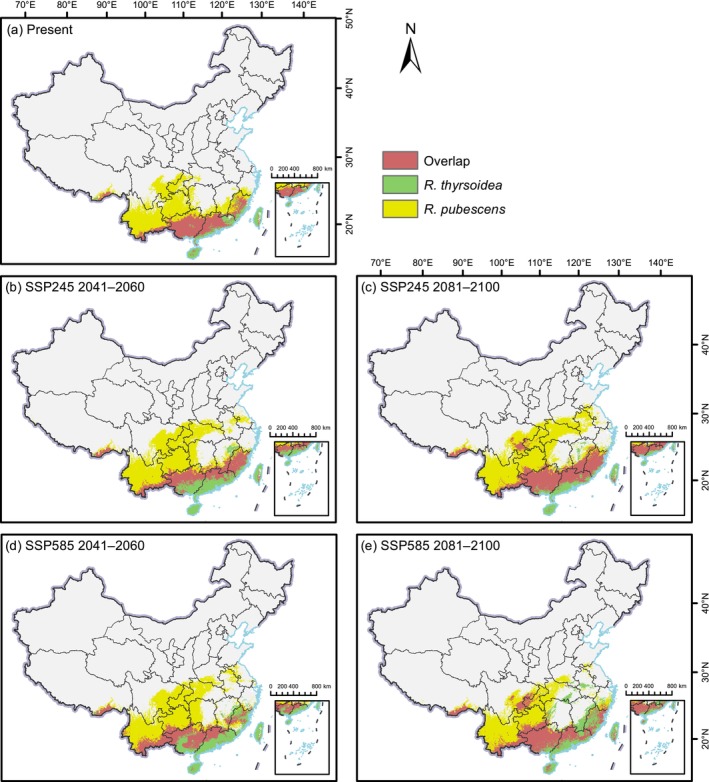
Spatial distribution of overlapping suitable habitats for *Reevesia pubescens* and *R*. *thyrsoidea* under different climate scenarios. (a) Present, (b) SSP245 2041–2060, (c) SSP245 2081–2100, (d) SSP585 2041–2060, (e) SSP585 2081–2100.

We also analyzed the spatial overlap of suitable habitats between *R. pubescens* and *R. thyrsoidea* under present and future climate scenarios (Figure 10). The results indicate a gradual increase in overlapping suitable areas toward the end of the century, suggesting a growing potential for spatial overlap under climate change. Currently, the total overlapping area is estimated at 34.50 × 10^4^ km^2^, accounting for 27.27% of the combined suitable habitats. During 2041–2060, the differences between scenarios are minor, with overlapping areas reaching 36.79 × 10^4^ km^2^ (22.75%) under the low‐emission SSP245 scenario, and 38.34 × 10^4^ km^2^ (23.53%) under the high‐emission SSP585 scenario. However, by 2081–2100, a more pronounced expansion is observed: overlapping areas increase to 52.20 × 10^4^ km^2^ (29.76%) under SSP245 and 50.68 × 10^4^ km^2^ (32.35%) under SSP585.

Geographically, the overlapping suitable habitats are mainly concentrated in most parts of Guangdong and Guangxi. Under future scenarios, particularly SSP245, substantial expansion of overlapping areas is also observed in Fujian. Smaller but notable overlapping patches are projected to appear in southeastern Sichuan, the southeastern border of Yunnan, and parts of southern Tibet (Figure 10). These findings suggest that, despite their distinct ecological preferences, both species may increasingly share suitable environments under future climate conditions, offering potential opportunities for integrated conservation efforts.

## 4 Discussion

### 4.1 Model Performance Evaluation

MaxEnt has become a widely accepted and robust tool for SDM, particularly when only presence data are available. It offers strong predictive performance and is capable of modeling complex ecological responses through regularization techniques that minimize overfitting (Phillips and Dudík 2008). In this study, we optimized model parameters based on ecological relevance and performance tests, which helped improve model reliability and reduce bias. Nevertheless, like all correlative models, MaxEnt involves several assumptions that introduce uncertainty. For instance, it assumes spatially uniform sampling and ecological niche stability over time. Although these assumptions are inherently constraining, the application of spatial filtering and rigorous variable selection reduces the influence of sampling bias and multicollinearity (Merow et al. 2013; Radosavljevic and Anderson 2014). Nevertheless, MaxEnt projections should be interpreted with caution, particularly when used for long‐term forecasting and conservation planning.

### 4.2 Divergence in Environmental Drivers and Niche Preferences

To clarify how current climate influences species distributions, we first evaluated the spatial patterns and key bioclimatic drivers associated with habitat suitability for *R. pubescens* and *R. thyrsoidea*. The results indicate that the two congeneric species differ markedly in their responses to climatic variables, revealing clearly differentiated ecological niche preferences. For *R. pubescens* , the most important predictor was precipitation of driest quarter (bio17), suggesting a strong sensitivity to drought conditions. In contrast, *R. thyrsoidea* was primarily influenced by temperature annual range (bio7), indicating a preference for thermal environments. This divergence is consistent with their current geographical distributions. *R. pubescens* is primarily found in the mountainous regions of SW China (e.g., Yunnan, Guizhou), where climates are characterized by pronounced seasonality, distinct dry periods, and heterogeneous topography (Qin et al. 2010; Wang et al. 2022). By contrast, *R. thyrsoidea* occurs mainly in the warm, humid lowlands of southeastern China (e.g., Guangdong, Guangxi), where annual temperature variation is relatively small and precipitation is both abundant and more evenly distributed across the year (Wang and Chen 2012).

Response curves further highlight these climatic preferences. *R. pubescens* shows high habitat suitability in areas with an annual mean temperature of approximately 20°C, a mean diurnal temperature range of ~7.5°C, and elevations around 2000 m. In contrast, *R. thyrsoidea* prefers warmer environments (mean annual temperature > 26°C), lower diurnal variation (< 5°C), and elevations near 500 m. Particularly notable is the difference in moisture requirements: *R. thyrsoidea* favors areas with nearly 100 mm of precipitation during the driest quarter—almost twice the threshold of *R. pubescens* —indicating lower drought tolerance and higher dependence on sustained humidity. These findings clearly indicate ecological niche differentiation between the two species. Such divergence among closely related taxa is often shaped by long‐term adaptation to different climatic regimes and ecological pressures (Anacker and Strauss 2014; Suarez‐Mota and Villasenor 2020).

Interestingly, topographic variables such as elevation and slope contributed minimally to the species distribution models for both species. This suggests that climatic factors, rather than terrain, are the dominant forces shaping current distributions. Similar patterns have been observed in other distribution modeling studies of tropical and subtropical species in South China, where topographic factors play a relatively minor role compared to climatic variables (Shi et al. 2023; Zhou et al. 2021). These results imply that future range shifts will likely be more strongly driven by changes in temperature and precipitation than by elevation or slope constraints.

### 4.3 Contrasting Future Range Dynamics and Conservation Implications

In terms of projected range shifts under future climate scenarios, our findings reveal distinct spatial dynamics between *R. pubescens* and *R. thyrsoidea*. Although both species are expected to experience range expansions, the scale and direction of these changes differ markedly. For *R. pubescens* , moderate expansion is projected under the SSP245 scenario. New suitable habitats are predicted to emerge in northern Hubei, southern Henan, and central Anhui. However, under the high‐emission SSP585 scenario, this expansion is notably weaker. Slight contractions are expected in lowland areas of southern Guangdong and Guangxi, suggesting that excessive warming may exceed the species' tolerance limits (Vasseur et al. 2014). In contrast, *R. thyrsoidea* shows a pattern of robust and continuous expansion under both scenarios, especially under SSP585. Substantial gains in extremely suitable areas are projected in regions such as Jiangxi, southeastern Sichuan, and even into Chongqing. These findings indicate that *R. thyrsoidea* may benefit more from warmer and wetter future climates.

The divergent responses of the two species can be attributed to differences in their climatic adaptations. *R. pubescens* , which is adapted to cooler montane environments, appears to have a narrower thermal tolerance. It performs best under moderate warming (e.g., SSP245), but may be physiologically constrained under extreme warming conditions. It is also sensitive to drought‐related changes. The projected increase in habitat suitability and northward expansion of *R. pubescens* in southwestern China closely align with the spatial distribution of optimal precipitation during the driest quarter (approximately 50–75 mm) across future climate scenarios (Supporting Information, Figure S7). This pattern is consistent with other montane species that are vulnerable under high‐emission scenarios due to limited upward migration space and narrow thermal ranges (Dullinger et al. 2012; Freeman et al. 2018). By contrast, *R. thyrsoidea* is better adapted to warm and humid environments. Its expansion under SSP585 suggests higher climatic tolerance and ecological plasticity. This trend is in line with previous studies indicating that tropical species often have greater potential to expand into cooler regions as temperatures rise (Wright et al. 2009).

These scenario‐specific differences suggest that long‐term conservation outcomes for *Reevesia* species will be strongly determined by future emission pathways, highlighting the importance of adaptive, forward‐looking conservation strategies. Regions that consistently remain suitable across both time periods and SSPs can be considered potential climate refugia and prioritized for long‐term in situ conservation, whereas areas showing suitability only under moderate warming scenarios may require more flexible management approaches to buffer against future climatic uncertainty. We also examined the extent to which their distributions may overlap in the future and the implications for conservation planning. The projected spatial overlap between *R. pubescens* and *R. thyrsoidea* reveals a modest but consistent increase under present and future climate scenarios, particularly by 2081–2100. Although the two species display distinct climatic preferences and occupy different ecological zones at present, their suitable habitats are expected to converge more in the future. Key overlap areas include Guangdong, Guangxi, Fujian, and southeastern Sichuan. These regions may serve as climate‐resilient habitats and should be prioritized in future conservation efforts for *Reevesia*. These zones are not only important for the persistence of *Reevesia* species under climate change but also hold broader conservation value. In contrast to narrowly distributed endangered plants, widespread yet ecologically significant genera like *Reevesia* play fundamental roles in ecosystem stability, carbon sequestration, and landscape integrity (Leung et al. 2018). Joint conservation of such habitats can help preserve functional diversity and ecosystem services, contributing to long‐term climate adaptation strategies (Porfirio et al. 2014; Tarjuelo et al. 2014). Furthermore, these zones may support species persistence and potential interspecific interactions (Ren et al. 2025). Targeted conservation and integrated land management within these zones can simultaneously advance multiple ecological objectives, including the protection of biodiversity, enhancement of carbon storage, and improvement of habitat connectivity (Soto‐Navarro et al. 2020; Xu et al. 2017).

## 5 Conclusions and Outlook

In this study, we used optimized MaxEnt models to simulate the current distributions and predict future distributions under different climate change scenarios of *R. pubescens* and *R. thyrsoidea*. The two congeneric species exhibit different ecological preferences and spatial responses. Specifically, *R. pubescens* is mainly distributed in montane regions of SW China and is most sensitive to dry‐season precipitation and temperature variability. It prefers elevations around 2000 m, moderate temperatures (~20°C), and about 50 mm of precipitation in the driest quarter. In contrast, *R. thyrsoidea* occupies lowland areas in southeastern China, favoring warm, humid conditions with low thermal seasonality, elevations below 500 m, and high moisture availability. The results indicate that climate exerts a stronger influence than topography in shaping their current distribution patterns. Future projections suggest that both species will expand their suitable habitats, but with different spatial patterns and magnitudes. *R. pubescens* is projected to undergo a moderate northward expansion under SSP245 (the low‐emission scenario), while *R. thyrsoidea* is expected to exhibit a marked and continuous expansion under both scenarios, particularly under SSP585 (the high‐emission scenario). Correspondingly, the total area of climatically suitable habitat for *R. pubescens* may increase by as much as 40%, while that for *R. thyrsoidea* may expand by more than 60%, contingent on the emission scenario considered. Suitable habitats for the two species are also expected to increasingly overlap, particularly in South China, including Guangdong, Guangxi, Fujian, and southeastern Sichuan by the end of the century. These overlap zones may offer important opportunities for joint conservation, as they support both species under future climate conditions. Looking ahead, broader regional modeling will become both feasible and necessary as species occurrence data from SE Asia (e.g., Vietnam, Laos, Thailand) become more comprehensive. Similarly, the inclusion of additional environmental drivers (e.g., soil properties, land cover, and biotic interactions) is expected to enhance the ecological realism and the predictive accuracy of species distribution models as relevant datasets become increasingly available. Overall, despite these current limitations, this study highlights species‐specific responses to climate change within *Reevesia* and provides important guidance for the development of adaptive conservation strategies across subtropical China.

## Author Contributions

**Xuanqi Liu:** conceptualization (equal), data curation (lead), investigation (equal), methodology (lead), validation (lead), visualization (lead), writing – original draft (lead), writing – review and editing (supporting). **Xia Meng:** conceptualization (equal), supervision (lead), validation (supporting), visualization (supporting), writing – review and editing (equal). **Minqiao Li:** visualization (supporting), writing – review and editing (supporting). **Zeyu Qin:** visualization (supporting), writing – review and editing (supporting). **Chen Li:** visualization (supporting), writing – review and editing (supporting). **Huasheng Huang:** conceptualization (lead), investigation (equal), methodology (supporting), project administration (lead), writing – review and editing (lead).

## Funding

This work was supported by the Starting Grant for Introduced Talents of Sun Yat‐sen University, the Fundamental Research Funds for the Central Universities, Sun Yat‐sen University (No. 24qnpy021), and the 2025 Basic and Applied Basic Research Project of Guangzhou Municipal Science and Technology Bureau (No. 2025A04J4384), and the Pearl River Recruitment Program of Talents of Guangdong Province, China (No. 2024QN11N111).

## Ethics Statement

The authors have nothing to report.

## Consent

The authors have nothing to report.

## Conflicts of Interest

The authors declare no conflicts of interest.

## Supporting information


**Data S1:** ece372934‐sup‐0001‐Supinfo01.docx.

**Data S2:** ece372934‐sup‐0002‐Supinfo02.xlsx.

## Data Availability

The curated species distribution data are accessible through the Global Biodiversity Information Facility (GBIF, http://www.gbif.org, http://doi.org/10.15468/dl.ycme2n) and the National Plant Specimen Resource Center (NPSRC, http://www.cvh.ac.cn/), and are also provided in the Supporting Information. The environmental variable data are available in the WorldClim database (version 2.1, http://www.worldclim.org).
