# Supplementary material for: Reevesia in a Warmer World: Mapping the Habitat Suitability of Its Two Representative Species (R. pubescens and R. thyrsoidea) in China
Source: Ecol Evol. 2026 Jan 12;16(1):e72934. doi: 10.1002/ece3.72934 (PMC12793777; doi:10.1002/ece3.72934)
Supplement: Supplementary file 1 — Data S1: ece372934‐sup‐0001‐Supinfo01.docx. [file ECE3-16-e72934-s002.docx]

Supporting Information for

*Reevesia* in a warmer world: Mapping the habitat suitability of its two representative species (*R. pubescens* and *R. thyrsoidea*) in China

Xuanqi Liu^a,b^, Xia Meng^a,b,*^, Minqiao Li^a,b^, Zeyu Qin^a,b^, Chen Li^a,b^, Huasheng Huang^a,b,*^

^a^School of Geography and Planning, Sun Yat-sen University, Guangzhou 510006, China

^b^Carbon-Water Observation and Research Station in Karst Regions of Northern Guangdong, School of Geography and Planning, Sun Yat-sen University, Guangzhou 510006, China

***Corresponding author:** Xia Meng, Huasheng Huang

**E-mail address:** mengx33@mail.sysu.edu.cn (X. Meng); huanghsh27@mail.sysu.edu.cn, buxushuang@gmail.com (H. Huang).

**Supporting Figures**


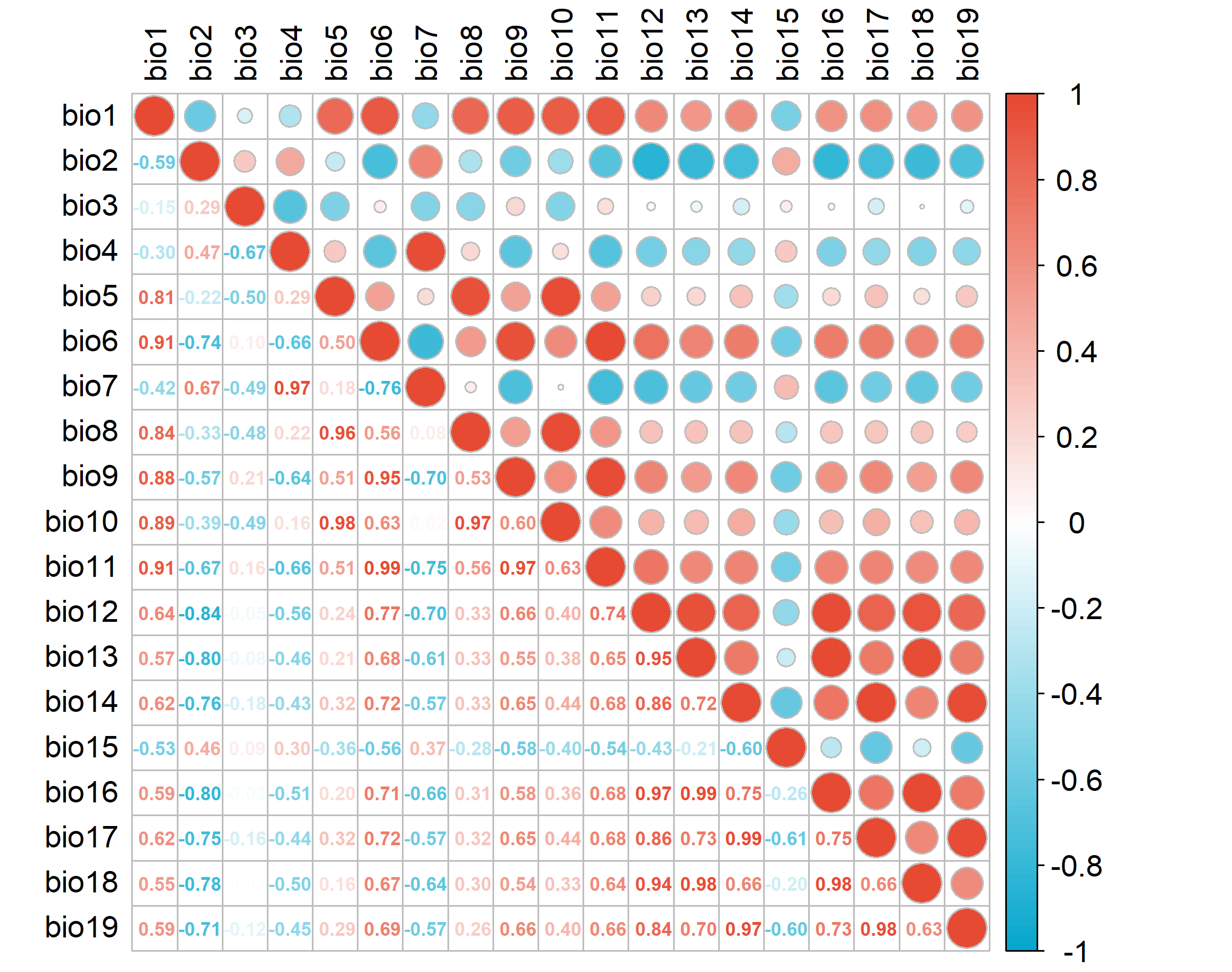


**Fig. S1.** Correlation matrix of the 19 bioclimatic variables. Positive correlations are shown in red and negative correlations in blue. The strength of the correlation is indicated by both the color intensity and the size of the circles, with larger and darker circles representing stronger relationships. Abbreviations: bio1, annual mean temperature; bio2, mean diurnal range; bio3, isothermality; bio4, temperature seasonality; bio5, max temperature of warmest month; bio6, min temperature of coldest month; bio7, temperature annual range; bio8, mean temperature of wettest quarter; bio9, mean temperature of driest quarter; bio 10, mean temperature of warmest quarter; bio11, mean temperature of coldest quarter; bio12, annual precipitation; bio13, precipitation of wettest month; bio14, precipitation of driest month; bio15, precipitation seasonality; bio16, precipitation of wettest quarter; bio17, precipitation of driest quarter; bio18, precipitation of warmest quarter; bio19, precipitation of coldest quarter.


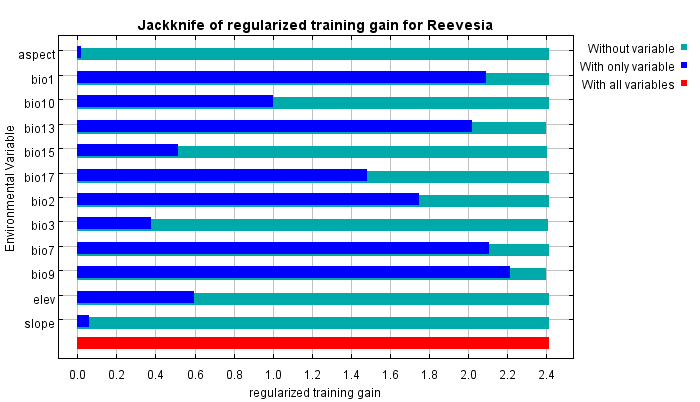


**Fig. S2.** Jackknife of regularized training gain for *Reevesia thyrsoidea* including aspect.


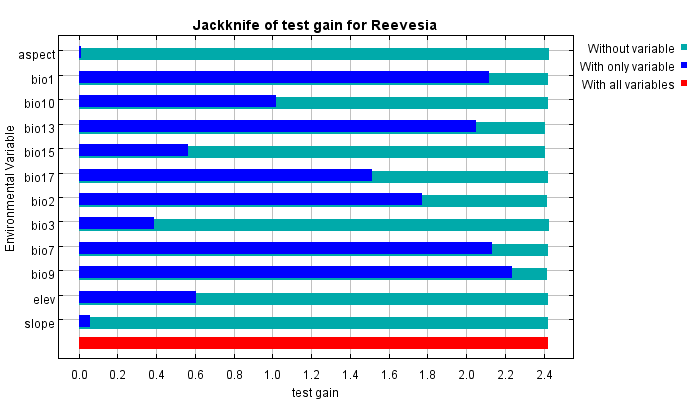


**Fig. S3.** Jackknife of test gain for *Reevesia thyrsoidea* including aspect.


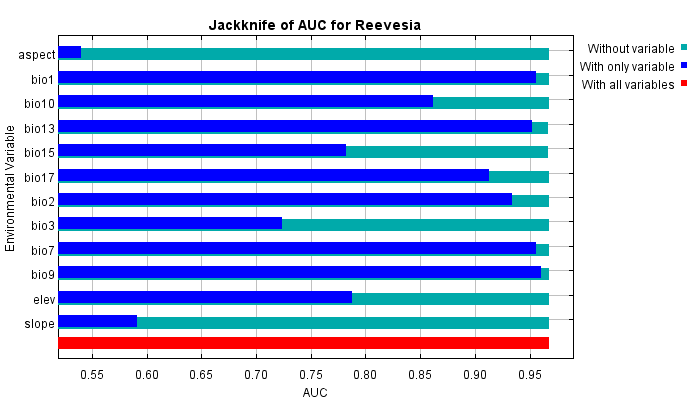


**Fig. S4.** Jackknife of AUC for *Reevesia thyrsoidea* including aspect.


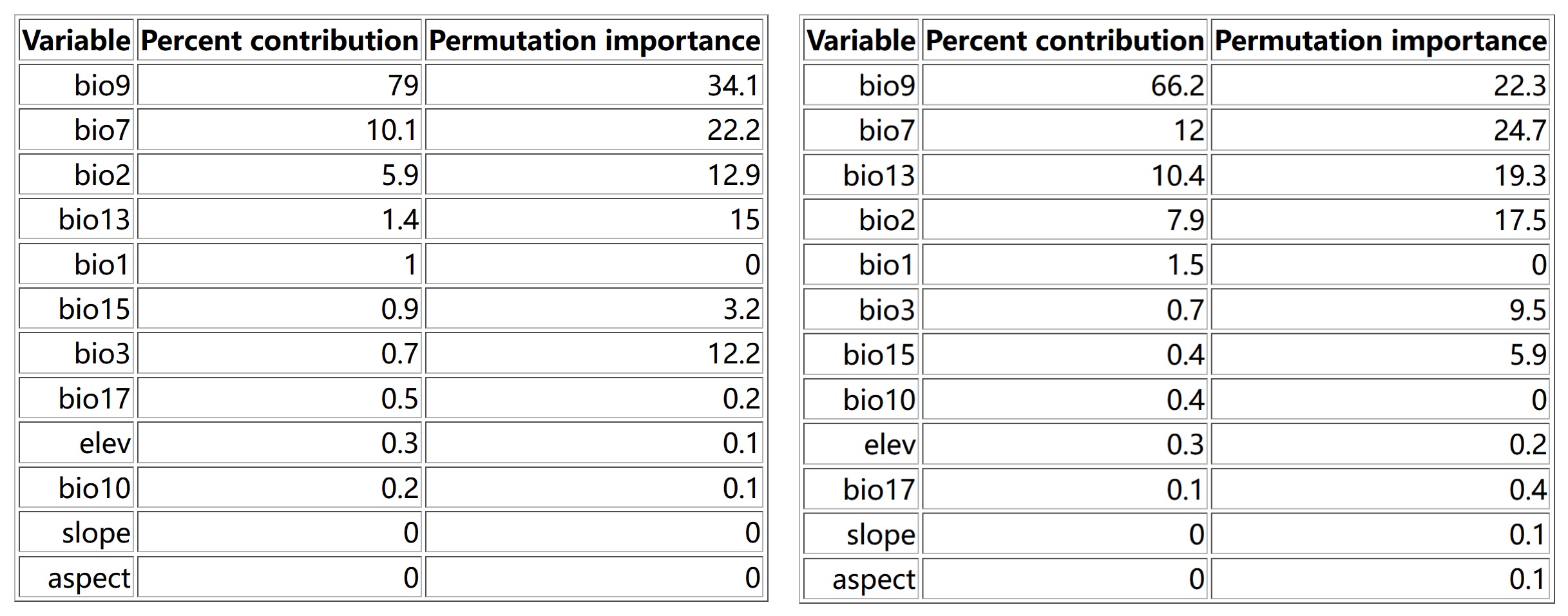


**Fig. S5.** Percent contribution and permutation importance of different environmental variables for *Reevesia* *thyrsoidea.* Values presented correspond to two representative runs selected from ten replicated model runs.


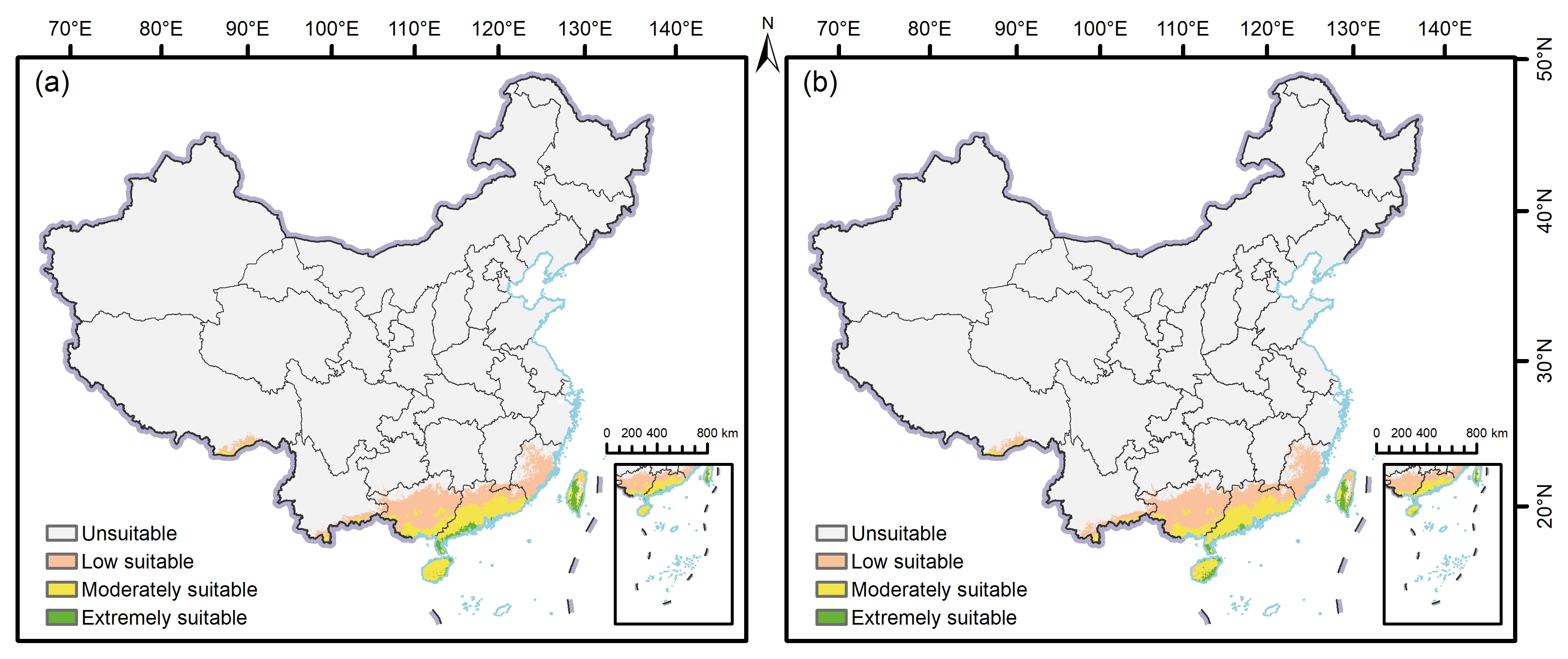


**Fig. S6.** Present-day modeling distribution of *Reevesia thyrsoidea* including aspect (a) and excluding aspect (b).


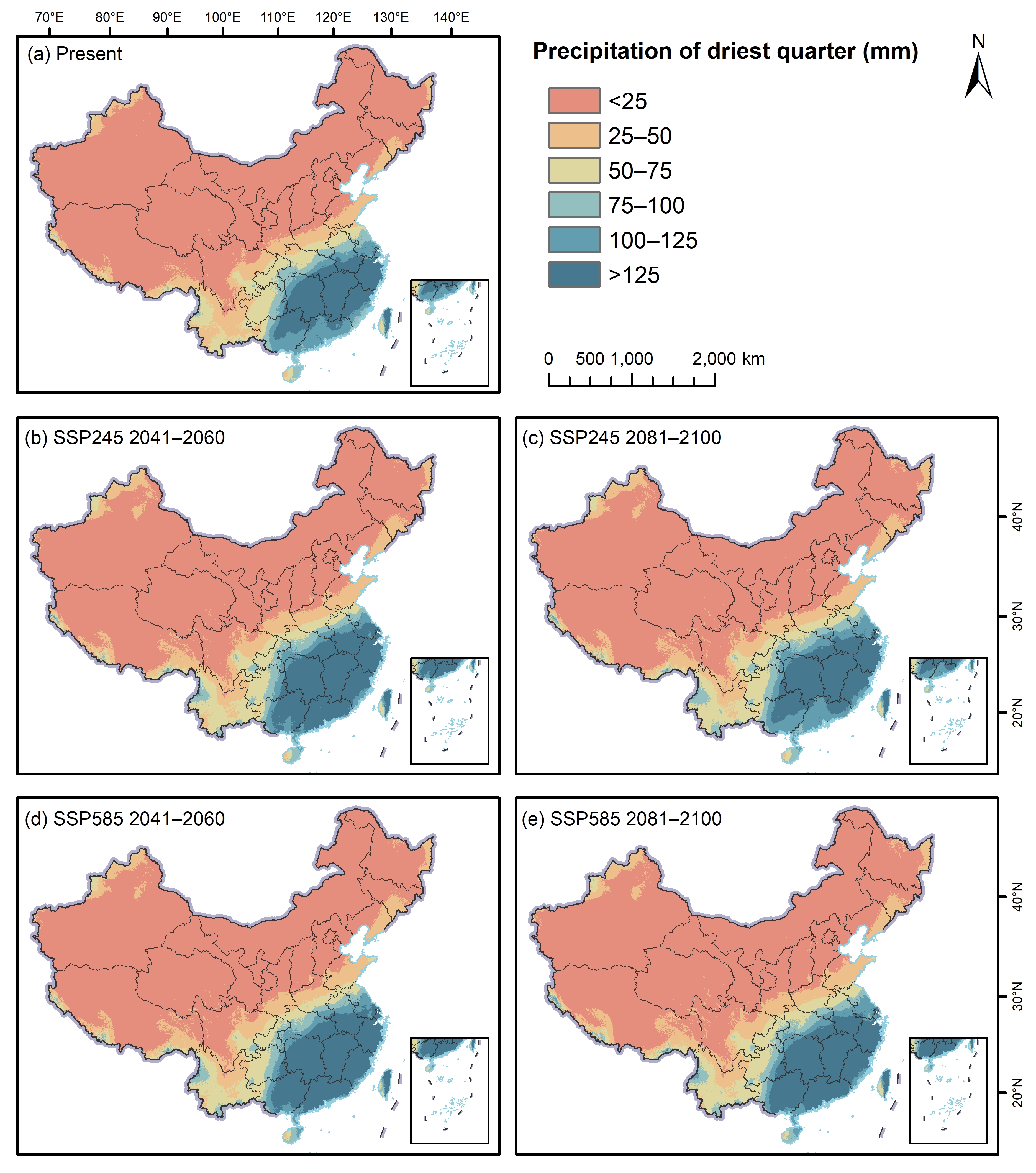


**Fig. S7.** Precipitation of the driest quarter (bio17) under the current climate and future SSP245 and SSP585 scenarios for two time periods (2041–2060 and 2081–2100).


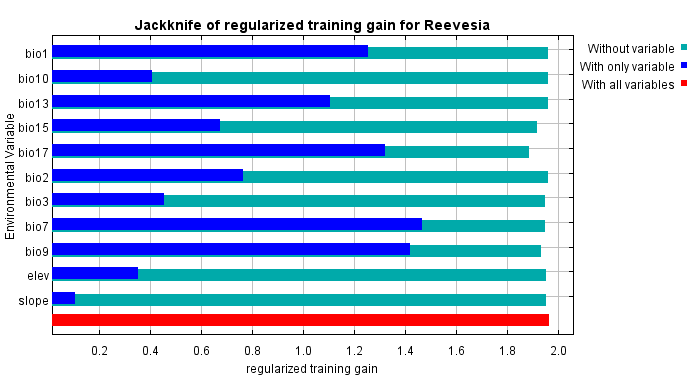


**Fig. S8.** Jackknife of regularized training gain for *Reevesia pubescens*.


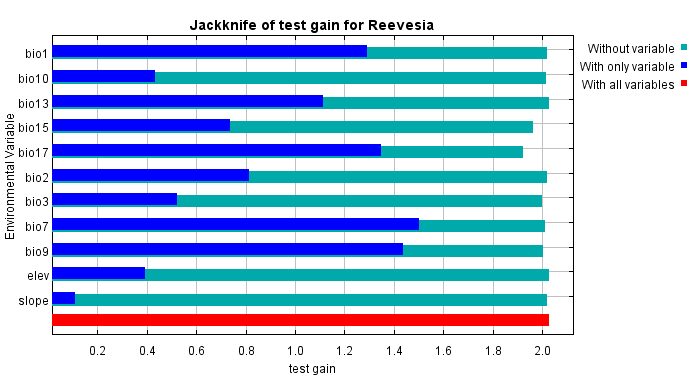


**Fig. S9.** Jackknife of test gain for *Reevesia pubescens*.


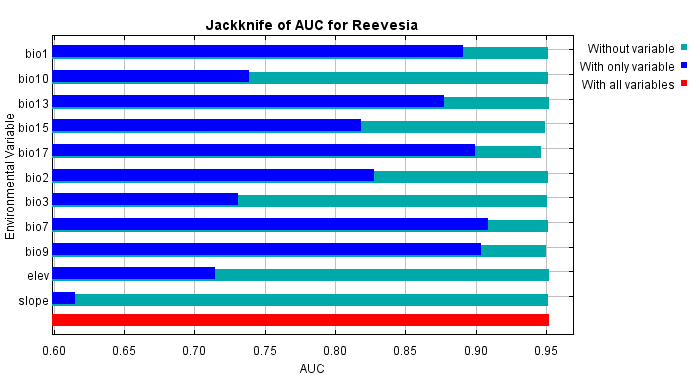


**Fig. S10.** Jackknife of AUC for *Reevesia pubescens*.


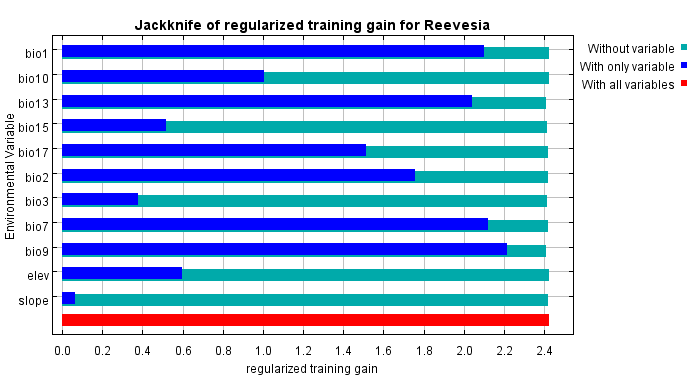


**Fig. S11.** Jackknife of regularized training gain for *Reevesia thyrsoidea.*


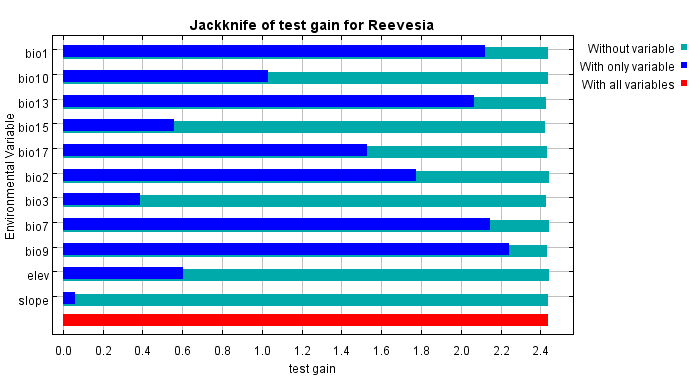


**Fig. S12.** Jackknife of test gain for *Reevesia thyrsoidea*.


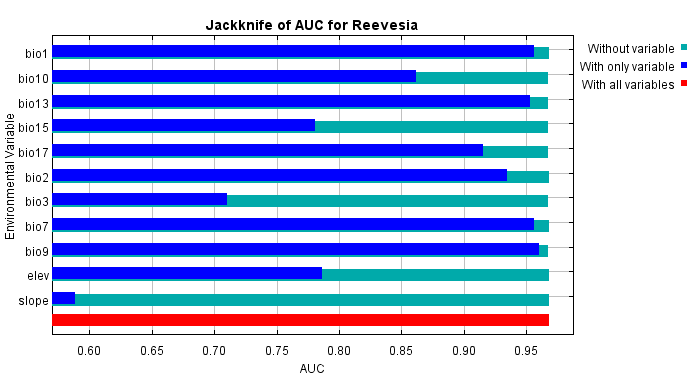


**Fig. S13.** Jackknife of AUC for *Reevesia thyrsoidea.*

**Supporting Text**

# **Jackknife test for *Reevesia thyrsoidea* including aspect** (**Figs. S2–S4)**

Figs. S2–S4 show the results of the jackknife test of variable importance. When used in isolation, bio9 produces the highest gain, indicating that it contains the greatest amount of unique explanatory information. In contrast, omission of bio13 results in the largest reduction in gain, suggesting that this variable contributes the most information not captured by the remaining predictors. The reported values represent averages across replicate model runs. Fig. S3 presents the same jackknife analysis based on test gain rather than training gain, and highlights that inferences regarding variable importance may differ when evaluated on independent test data. The corresponding jackknife test using AUC on the test data is shown in Fig. S4.

# **Jackknife test for *Reevesia pubescens* and *R. thyrsoidea*** (**Figs. S8****–S13)**

For *Reevesia pubescens*, Fig. S8 presents the results of the jackknife test of variable importance based on training gain. Among the predictors, bio7 yields the highest gain when used in isolation, indicating that it provides the greatest explanatory power on its own. The environmental variable that decreases the gain the most when it is omitted is bio17, which therefore appears to have the most information that isn't present in the other variables. Values shown are averages over replicate runs. Fig. S9 shows the same jackknife test, using test gain instead of training gain. Note that conclusions about which variables are most important can change, now that we're looking at test data. The same jackknife test using AUC on test data is Fig. S10.

Similarly, for *R. thyrsoidea*, Fig. S11 displays the jackknife test of variable importance on training gain. In this case, bio9 achieves the highest gain when used alone, whereas exclusion of bio13 leads to the greatest decline in gain, again indicating that it contributes the most non-redundant information. Values are averaged across replicate runs. The same analysis using test gain is shown in Fig. S12, where the ranking of variable importance changes when assessed with test data. The jackknife test using test AUC is provided in Fig. S13.
